# Physical crowding truncates intrinsic Lévy-like motility into caste-specific movement regimes in social termites

**DOI:** 10.1186/s40462-026-00646-w

**Published:** 2026-04-03

**Authors:** Zhuang-Dong Bai, Jia-Xu Han, Nan Ye, Chao Wang, David Sillam-Dussès, Rui-Wu Wang

**Affiliations:** 1 Ministry of Education Key Laboratory for Ecology of Tropical Islands, Key Laboratory of Tropical Animal and Plant Ecology of Hainan Province, College of Life Sciences, Hainan Normal University, Haikou, China; 2 The First Affiliated Hospital, College of Life Science and Technology, Jinan University, Guangzhou, Guangdong 510632 China; 3 Center for Materials Synthetic Biology, Shenzhen Institute of Synthetic Biology, Shenzhen Institute of Advanced Technology, Chinese Academy of Sciences, Shenzhen, 518055 China; 4 School of Life Science and Technology, Northwestern Polytechnical University, Xi’an, 710072 China; 5 Laboratory of Experimental and Comparative Ethology, LEEC, UR4443, University Sorbonne Paris Nord, Villetaneuse, France; 6 College of Life Sciences, Zhejiang University, Hangzhou, 310058 China

**Keywords:** Truncated Lévy walk, *Reticulitermes labralis*, Physical crowding, Division of labor, Caste-specific resilience

## Abstract

Lévy-like movement patterns, characterized by heavy-tailed step-length distributions, are often considered a highly efficient strategy for solitary searching. While recent studies have established that isolated termite workers possess intrinsic Lévy-like motility templates, how these scale-free patterns differ across functionally distinct castes—and how they adapt to the extreme physical crowding typical of natural subterranean galleries remains unresolved. Here, using high-resolution tracking of the subterranean termite *Reticulitermes labralis* across group sizes from solitary individuals (*N* = 1) to high jammed crowds (*N* = 1,000), we reveal a fundamental motility asymmetry between castes. Building upon established worker baseline, we demonstrate that these intrinsic behavioral templates are highly heterogeneous: although solitary individuals of both castes intrinsically exhibit Lévy-like motion, workers maintain a significantly stronger super-diffusive baseline compared to queens, reflecting an innate predisposition for extensive exploration. As social density increases, physical blocking (steric repulsion) universally truncates trajectories toward multi-phasic Brownian dynamics. However, this truncation is functionally divergent: workers retain greater mobility resilience, preserving exploration efficiency even under crowding, whereas queens rapidly succumb to physical entrapment. Integrating empirical data with an Agent-Based Model, we show that this transition emerges naturally from pervasive physical constraints. Our findings suggest that the interplay between caste-specific intrinsic templates and uniform physical blocking spontaneously organizes the colony’s division of labor: workers are tuned for efficient exploration, while queens are physically constrained to become static interaction hubs.

**Supplementary Information:**

The online version contains supplementary material available at 10.1186/s40462-026-00646-w.

## Introduction

Optimal movement strategies are fundamental to individual fitness and ecosystem functioning. In resource-scarce environments, super-diffusive movement patterns, particularly Lévy walks, have been identified as an evolutionary optimal strategy for maximizing search efficiency [1–5]. Theoretically defined by a power-law distribution of step lengths (*P*(*l*) ~ *l*^−*µ*^ with 1 < *µ* ≤ 3) within a bounded domain, this statistical pattern has been documented across a diverse range of solitary taxa, from bacteria and T-cells to marine predators and human hunter-gatherers [6–9]. However, the universality of the Lévy walk foraging hypothesis has been increasingly questioned. Recent theoretical and empirical studies have demonstrated that the optimality of Lévy searches is highly conditional, depending on factors such as target density and whether foraging is destructive or non-destructive [10]. Furthermore, advanced physical analyses have identified a range of non-Lévy behaviours in diverse biological and physical systems, suggesting that super-diffusive patterns may often be transient or replaced by more complex multi-state dynamics under specific environmental constraints [11–13].

For social animals, particularly eusocial insects like ants and termites, movement occurs within a fundamentally different context: high-density crowded [14]. In these systems, individuals are constantly subjected to physical collisions and social interactions, which can theoretically inhibit long-range displacements and enforce highly restricted, localized dynamics [15–17]. While some studies suggest that social interactions can induce Lévy-like patterns via information transfer or quorum sensing [18–20], others argue that crowding acts as a source of “noise” that disrupts optimal trajectories, leading to traffic jams or glassy dynamics [21, 22]. This raises a fundamental question: how do colony members maintain functional dispersion when their physical mobility is severely constrained by nestmate density?

Subterranean termites (*Reticulitermes* spp.) offer an ideal model to resolve this dilemma. Unlike open-air foragers, termites operate in confined, dark tunnel systems where physical crowding is unavoidable and continuous [23, 24]. Furthermore, termite colonies exhibit extreme division of labor between morphologically distinct castes: workers, which perform extensive foraging and brood care, and queens, which serve as the reproductive center [25, 26]. This functional dichotomy suggests that different castes may have evolved distinct movement baselines or differential sensitivities to social crowding, workers retaining high mobility for exploration, while queens adopt localized patterns to maintain colony cohesion.

Here, building on this functional dichotomy, we combined high-resolution tracking of *Reticulitermes labralis* with statistical physics modeling to investigate the density-dependent origins of movement patterns. We systematically varied group sizes from solitary individuals (*N* = 1) to high-density crowds (*N* = 1,000) for both queens and workers. Using this design, we tested whether distinct castes possess heterogeneous intrinsic movement templates, and how these templates respond to increasing social density. Specifically, building upon the known modulatory effects of active social interactions [27, 28], we investigated whether pervasive physical constraints (steric blocking) at extreme densities are mechanistically sufficient to drive the caste-specific truncation of these scale-free templates. By integrating empirical trajectory analysis [29] with an Agent-Based Model (ABM), we demonstrate that the transition from ballistic to tortuous motion is primarily driven by physical blocking. Crucially, we reveal a caste-specific functional trade-off: workers intrinsically possess super-diffusive capabilities and exhibit greater resilience to crowding, whereas queens rapidly succumb to physical entrapment. These findings provide a mechanistic framework for understanding how physical constraints and biological adaptations co-shape the collective movement of social colonies.

## Materials and methods

### Termite collection and maintenance

Five colonies of *Reticulitermes labralis* were collected from decayed wood in the northern Qinling Mountains, Xi’an, Shaanxi Province, China (108°46′E, 34°00′N) in August 2023. Colonies were maintained in ventilated plastic boxes (80 × 50 × 40 cm) containing moist pine at 25 °C and 70% relative humidity (RH) for one week to allow acclimation.

To induce the differentiation of supplementary reproductievs, approximately 1,000 individuals from each colony were established in 9-cm diameter Petri dishes (five replicates per colony) provisioned with damp filter paper as food and moisture sources. Caste identity was confirmed based on morphological traits, including body pigmentation and abdominal shape. After one month, neotenic queens differentiated within each group, identified by characteristic head pigmentation and slight abdominal enlargement [30–32].

For movement analysis, experimental groups were established in 9-cm Petri dishes lined with moist filter paper. Four group sizes were tested: (i) Solitary (*N* = 1): one randomly selected queen or worker; and (ii) Groups (*N* = 100, 200, 1,000): consisting of mixed castes to mimic natural colony composition. Specifically, the *N* = 100 group comprised 94 workers, 4 soldiers, 1 queen, and 1 king; the *N* = 200 group comprised 190 workers, 8 soldiers, 1 queen, and 1 king; the *N* = 1,000 group comprised 958 workers, 40 soldiers, 1 queen, and 1 king. All groups were maintained under controlled conditions (25 °C, 70% RH, 14 h light/10 h dark) and acclimated for 24 h prior to behavioral recording to mitigate handling stress.

### Movement recordings

To enable individual identification, the abdomen of each focal queen or worker was marked with a small spot of non-toxic white glue mixed with dye following the protocol of Marins et al. [33]. Marked individuals were allowed to dry for 24 h to minimize behavioral artifacts. Groups were then transferred sequentially to a recording arena and allowed to acclimate for one hour. Movements were recorded continuously for 51 min using a Sony AX100E camera (1920 × 1080 pixels) positioned overhead under uniform illumination. Video files were downsampled to 5 frames s⁻¹ for analysis in EthoVision XT (Noldus Information Technology, Wageningen, The Netherlands) The X–Y coordinates of the body centroid were extracted for subsequent processing (Figure S1). In total, 25 complete recordings were analyzed, covering five biological replicates across five experimental conditions: solitary queens (*N* = 1), solitary workers (*N* = 1), and mixed groups of *N* = 100, 200, 1,000 individuals. Each recording yielded approximately 15,000 validated positional data points. In high-density groups, occasional short interruptions occurred due to individuals climbing the dish walls. These intervals results in missing coordinates, which were linearly interpolated during pre-processing to ensure data integrity without biasing the trajectory analysis.

### Quantification of short-term path tortuosity

To assess movement tortuosity while mitigating boundary effects imposed by the circular arena, we calculated the Straightness Index using a sliding window approach. For each trajectory, a temporal window of 5 s (25 frames at 5 Hz) was moved stepwise with a 1-second increment. Within each window, the straightness index (S) was calculated as the ratio of the net displacement (D, Euclidean distance between the first and last points) to the total path length (L, sum of inter-frame distances): S = D/L. An index close to 1 indicates ballistic, straight-line motion, while values approaching 0 indicate highly tortuous paths. The mean straightness index across all windows was calculated for each individual to represent its baseline movement characteristic.

### Analysis of velocity correlations

To verify the independence of successive steps and assess the validity of the semi-Markov process assumption, we calculated the velocity autocovariance function (VACF) for both castes across all density conditions [34]. The VACF, *C*_*v*_(*τ*), quantifies the correlation between velocity vectors *v*(*t*) separated by a time lag *τ*, defined as:

$$Cv(\tau) = {{\left\langle {v(t) \cdot v(t + \tau)} \right\rangle } \over {\left\langle {{{\left| {v(t)} \right|}^2}} \right\rangle }}$$

Where $\left\langle \cdot \right\rangle$ denotes the ensemble average over all trajectories. A rapid decay of *C*_*v*_(*τ*) to zero indicates that directional memory is short-lived, implying that reorientations effectively randomize direction.

### Step-length extraction and analysis

To characterize the statistical properties of termite movement, step lengths were extracted from the raw 2D trajectories using the one-dimensional projection method described by Humphries et al. (2013) [29]. Briefly, the two-dimensional movement path was decomposed into independent projections along the *x* and *y* axes. A step was defined as the distance traveled between two consecutive reversal points along each projected axis. This method avoids the artifact of autocorrelation inherent in fixed-time interval sampling and allows for the detection of natural movement pauses and reorientations. Prior to analysis, trajectories were downsampled to 1 Hz to minimize high-frequency measurement noise. A minimum step length threshold (*l*_*min*_ = 0.5 mm) was applied to filter out micromovements associated with body wobbling or tracking jitter. The maximum step length (*l*_*max*_) was defined as the largest observed step in the sample.

### Maximum likelihood estimation and model selection

We fitted three competing statistical models to the empirical step-length distributions to identify the underlying movement dynamics: (i) Truncated Power-Law (TPL): representing Lévy walks within a bounded arena [35, 36]. (ii) Truncated Exponential (TE), representing Brownian motion constrained by physical boundaries [37, 38]. (iii) Bi-Exponential (Bi-Exp): representing a composite Brownian motion consisting of two distinct motility modes (e.g., intensive local search and extensive relocation) [39–41]. The probability density functions (PDFs) for these models are defined as follows:

(i) Truncated Power-Law (TPL):

$$\:p\left(l\right)={C}_{TPL}\cdot\:{l}^{-\mu\:},\mathrm{\:\:\:for\:}{l}_{min}\le\:l\le\:{l}_{max}$$

The step-length distribution was modeled using a power-law density with a hard upper-bound truncation, defined over the domain [*l*_*min*_, *l*_*max*_]. Where *µ* is the scaling exponent and $\:{C}_{TPL}=\frac{\mu\:-1}{{l}_{min}^{1-\mu\:}-{l}_{max}^{1-\mu\:}}$ is the normalization constant. This resolve the infinite variance paradox of pure Lévy stable densities in finite physical systems [42, 43].

(ii) Truncated Exponential (TE):

$\:p\left(l\right)={C}_{TE}\cdot\:{e}^{-\lambda\:l}\mathrm{,\:\:\:}\:\mathrm{for\:}{l}_{min}\le\:l\le\:{l}_{max}$ Where *λ* is the exponential decay rate and $\:{C}_{TE}=\frac{\lambda\:}{{e}^{-\lambda\:{l}_{min}}-{e}^{-\lambda\:{l}_{max}}}$ is the normalization constant.

(iii) Bi-exponential (Bi-Exp):

$$\:p\left(l\right)=w{\lambda\:}_{1}{e}^{-{\lambda\:}_{1}l}+(1-w){\lambda\:}_{2}{e}^{-{\lambda\:}_{2}l}$$

Where *w* is the weighting factor (0 ≤ *w* ≤ 1) between two exponential components with rates *λ*_1_ and *λ*_2_.

Model parameters (scaling exponent *µ*, decay rates *λ*, and mixing weight *w*) were estimated using Maximum Likelihood Estimation (MLE). The negative log-likelihood functions were minimized using the L-BFGS-B optimization algorithm implemented in the *scipy.optmize* library (Python 3.12). To select the best-fitting model, we calculated the Akaike Information Criterion (AIC) for each candidate model:

  

$$AIC=2k - 2\ln (\mathcal{L})$$

Where *k* is the number of parameters and $\mathcal{L}$ is the maximum likelihood value. Akaike weights (*w*_*AIC*_) were derived to quantify the relative probability that a specific model is the best approximation of the data among the candidate set. A model was considered the best fit if its Akaike weight exceeded 0.9. In cases where no single model reached this threshold, the model with the lowest AIC value was identified as the best descriptor. All analyses were performed using custom Python scripts utilizing the *NumPy* and *Pandas* libraries.

To visualize the goodness-of-fit, we plotted the complementary cumulative distribution functions (CCDF) of the empirical data against the theoretical curves of the three competitive models. It is important to note that while the probability density function (PDF) of a truncated power-law appears as a straight line on a log-log plot, its theoretical CCDF exhibits a characteristic downward curvature that drops precipitously to zero as the step length approaches the hard upper bound (*l*_*max*_). Finally, we summarized the prevalence of each movement behaviour across castes and group sizes by calculating the proportion of individuals best fitted by each model (detailed statistics in Table S1).

### Agent-based simulation of social crowding

To test the hypothesis that the observed transition from ballistic to tortuous movement is driven by physical blocking, we developed a spatial Agent-Based Model (ABM). The simulation environment was defined as a two-dimensional circular arena with a radius *R* = 45 mm, matching the experimental petri dish.

Intrinsic movement rules: Agents were modeled as persistent random walkers with an intrinsic drive to perform Lévy walks. Step lengths were drawn from an unbounded power-law distribution. We set the intrinsic scaling exponent *µ*_*int*_ as 1.35, consistent with the empirically observed ballistic behaviour of solitary queens. The minimum step length was set to 1.0 mm.

Physical and social constraints: Two types of truncation mechanisms were implemented to simulate environmental constraints: (1) Boundary truncation. If a generated step vector projected the agent outside the arena radius (*R*_*arena*_ - *R*_*agent*_, where *R*_*agent*_ = 1.0 mm), the step was truncated at the collision point with the wall, simulating a stop-at-boundary interaction. (2) Social blocking (density-dependent truncation). To mimic physical crowding, we implemented a stochastic collision probability that increases with group size (*N*). The probability of an agent successfully completing a step of length without social interruption was modeled as a Poisson-like process:

$$\:{P}_{success}\left(l\right)={exp}(-k\left(N\right)\cdot\:l)$$

Where *k*(*N*) is a density-dependent interruption rate scaling with $\:\sqrt{\mathrm{N}}$. If a collision occurred (determined stochastically), the intended long step was aborted and replaced by a short stagnation displacement, simulating the loss of momentum due to physical obstruction.

Parameter estimation and comparison: Simulations were performed for group sizes $\:\mathrm{N}\in\left\{\mathrm{1,\:100,\:200,\:1000}\right\}$. For each density, we ran 5 independent trials (each consisting of *N* agents moving for 15,00 steps). The resulting simulated step-length distributions were analyzed using the same Maximum Likelihood Estimation pipeline applied to the experimental data. Specifically, we fitted the Truncated Power-Law model to the simulated trajectories to derive an effective scaling exponent (*µ*_*eff*_). The trend of *µ*_*eff*_ across densities was then compared with experimental observations to validate the physical blocking hypothesis.

### Calculation of encounters, movement distance, and spatial exploration

To quantify social interaction, movement performance, and spatial exploration, we analyzed the recordings of color-marked queens and workers in 100-individual groups. 51min video segments were analyzed at 5 fps. Social encounters were define as physical contacts between the antennae of the focal (marked) individual and any body part of a nestmate. To distinguish distinct events, consecutive contacts separated by less than 1 s were pooled into a single encounter event. The total frequency of encounters was calculated for each focal individual and averaged across five replicate colonies.

Movement distance was computed as the cumulative Euclidean distance traveled by the centroid of the focal individual over the observation period. To assess spatial exploration, the circular arena was discretized into a square grid of 1 × 1 mm cells. The spatial coverage rate was calculated as the ratio of unique cells visited by the focal termite (defined as the grid cell containing the individual’s centroid) to the total number of accessible cells within the arena. This metric serves as a proxy for exploratory efficiency, independent of total distance traveled.

## Results

### Density-dependent transition from ballistic to diffusive movement

**Fig. 1:**
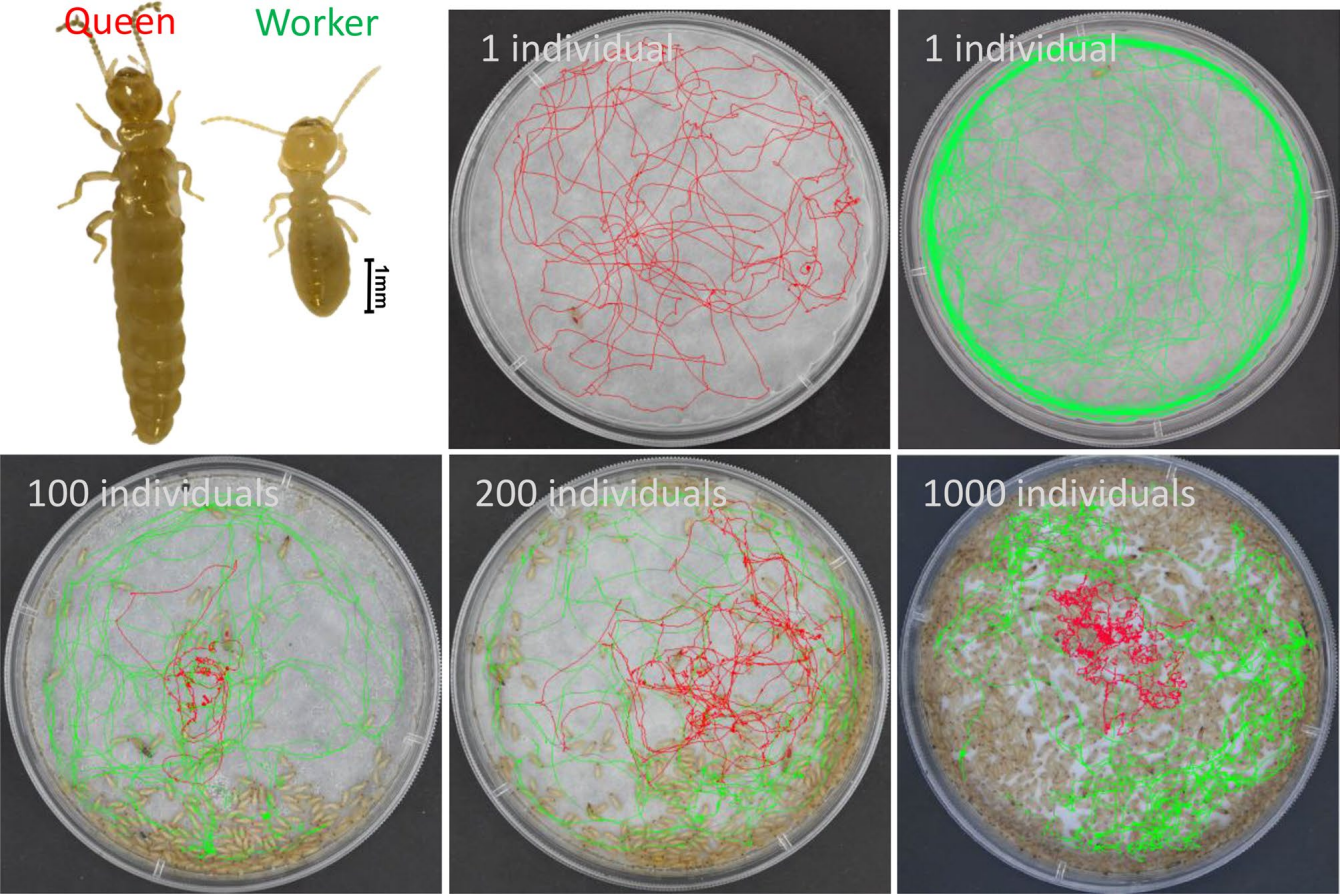
Morphological contrast and representative movement trajectories of reproductive queens and workers across social densities in termites. The upper left panel shows the marked size difference between a queen (left) and a worker (right). The remaining panels display movement trajectories recorded in experimental arena (90 mm diameter) at four distinct group sizes: solitary conditions (*N* = 1) and social groups of 100, 200, and 1,000 individuals. Red curves indicate the movement paths of queens, and green curves indicate the movement paths of workers

**Fig. 2:**
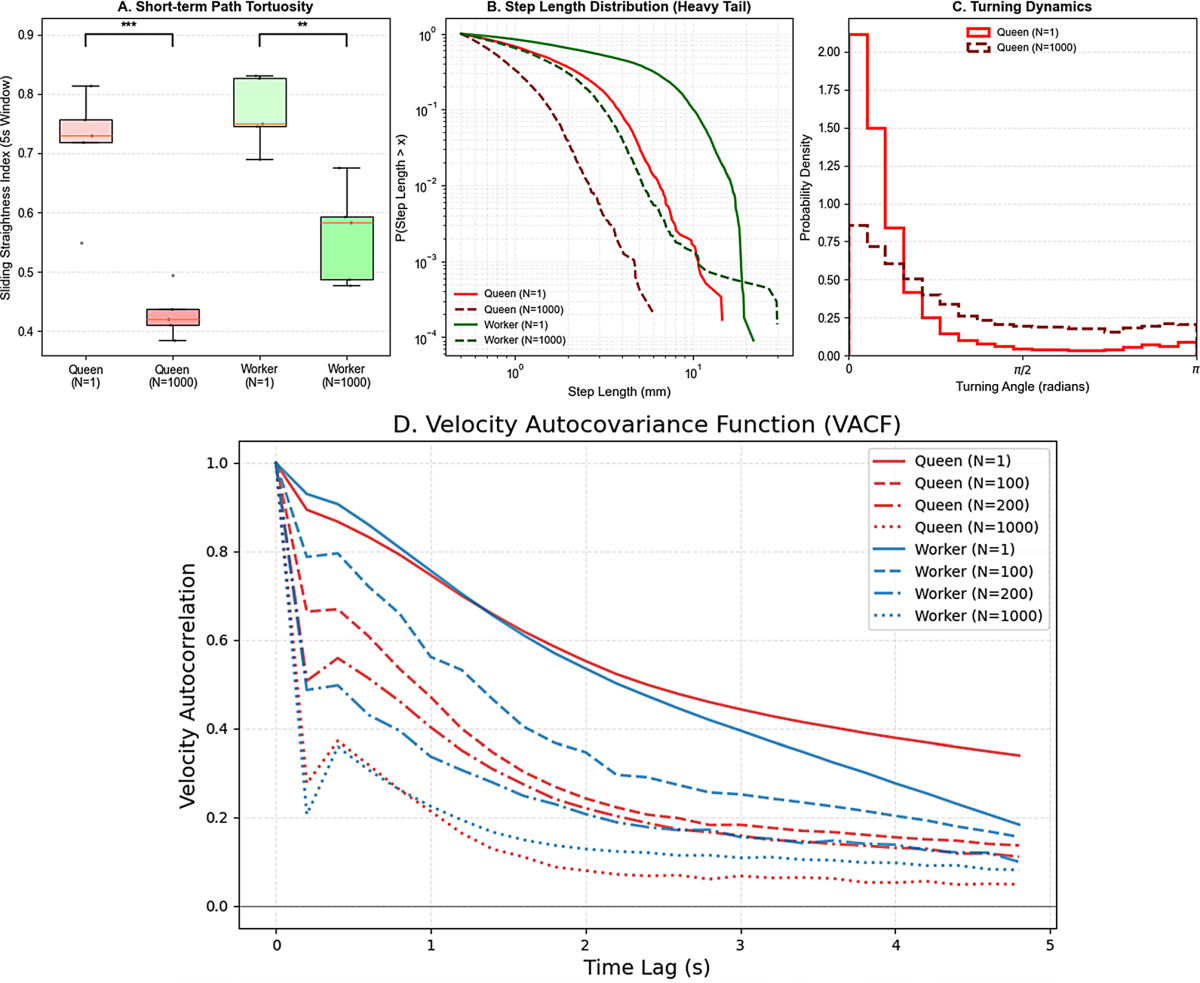
Quantitative comparison of movement dynamics and velocity autocorrelation between solitary and crowded conditions. (**A**) The Straightness Index (net displacement divided by total path length) significantly decreases at high density (*N* = 1,000) for both queens and workers compared with solitary conditions (*N* = 1; *** *P* < 0.001, ** *P* < 0.01), indicating the transition from ballistic to tortuous paths. (**B**) Complementary cumulative distribution functions (CCDFs) of step lengths plotted on a log-log scale. Solitary individuals (solid lines) exhibit heavy tails indicative of frequent long-range displacements, whereas crowed individuals (dashed lines) show truncated tails, reflecting the physical inhibition of long steps. (**C**) Distribution of turning angles. Solitary queens (solid red line) show a strong preference for small angles (forward persistence), while crowded queens (dashed dark red line) exhibit a flattened distribution, indicating frequent and random directional changes driven by collisions. (**D**) Velocity Autocovariance Function (VACF) plotted against time lag for queens and workers across varying group sizes. The VACF quantified the directional persistence of movement. A slow decay in autocorrelation (observed at *N* = 1) indicates persistent, ballistic-like motion characteristic of Lévy walks. In contrast, a rapid decay to zero (prominent at *N* = 1,000) reflects the quick randomization of movement direction

To investigate how social density shapes termite movement, we tracked the trajectories of queens and workers across four group sizes (*N* = 1, 100, 200, 1,000) in a standardized arena (Fig. 1). Analysis of the trajectories revealed a striking transformation: solitary individual (*N* = 1) performed long, straight-line displacements characteristic of ballistic motion, whereas individuals in high-density groups (*N* = 1,000) exhibited highly tortuous, localized paths. This qualitative shift was quantified using the straightness index, where values close to 1 indicate straight trajectories. Under solitary conditions, both queens and workers maintained high straightness values. In contrast, in high-density groups, straightness decreased significantly for both queens (*t*₈ = -5.940, *P* < 0.001) and workers (*t*₈ = -4.517, *P* < 0.01; Student’s t-test; Fig. 2A).

These changes in path geometry were underpinned by systematic shifts in step-lengh statistics and turning dynamics. Complementary cumulative distribution functions of step lengths revealed heavy-tailed distributions under solitary conditions. At high density, these distributions became markedly truncated (Fig. 2B). Furthermore, turning-angle distributions revealed a density-dependent loss of directional control. Solitary queens showed a strong bias toward small turning angles, indicative of forward persistence, whereas crowded queens exhibited a flattened turning-angle distribution, reflecting frequent, randomized reorientations associated with physical collisions (Fig. 2C). Finally, velocity autocovariance analysis provided a dynamical signature of this transition. Under solitary conditions, the velocity autocovariance functions (VACF) decayed slowly with increasing time lag (Fig. 2D). As group size increased, the VACF decayed progressively faster, particularly in queens. Workers retained detectable short-term velocity correlations over a broader range of densities, highlighting a caste-specific resilience to social crowding compared to the highly sensitive movement patterns of queens.

### Statistical collapse of heavy-tailed step distributions

**Fig. 3:**
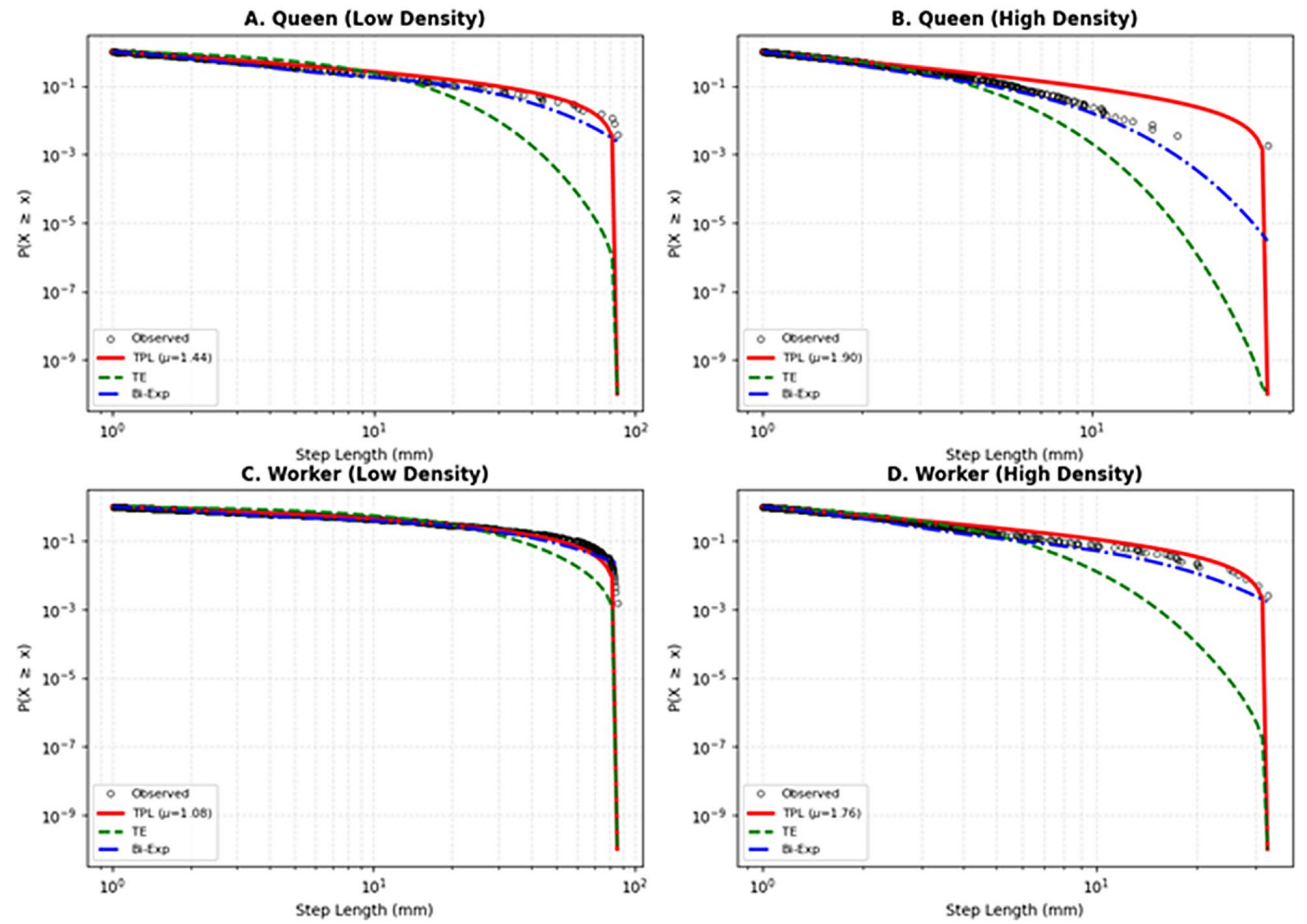
Step-length distributions reveal a density-dependent transition in movement dynamics. Complementary cumulative distribution functions (CCDF) of step lengths plotted on log-log scales for representative individuals: (**A**) Queen in isolation (*N* = 1); (**B**) Queen in a high-density group (*N* = 1,000); (**C**) Worker in isolation (*N* = 1); and (**D**) Worker in a high-density group (*N* = 1,000). Black circles represent empirical data. Colored lines represent the best-fit curves for three competitive models: Truncated Power-law (TPL, red solid line), Truncated Exponential (TE, green dashed line), and Bi-exponential (Bi-Exp, blue dot-dashed line). Note that in isolated conditions (**A**, **C**), the data follow a pattern characteristic of Lévy walks and are best fitted by the TPL model. In contrast, under social crowding (**B**, **D**), the distributions exhibit a distinct downward curvature (multi-phasic decay) and are best described by the Bi-exponential model, indicating a shift from intrinsic Lévy searches to interaction-mediated multi-phasic movements

**Fig. 4:**
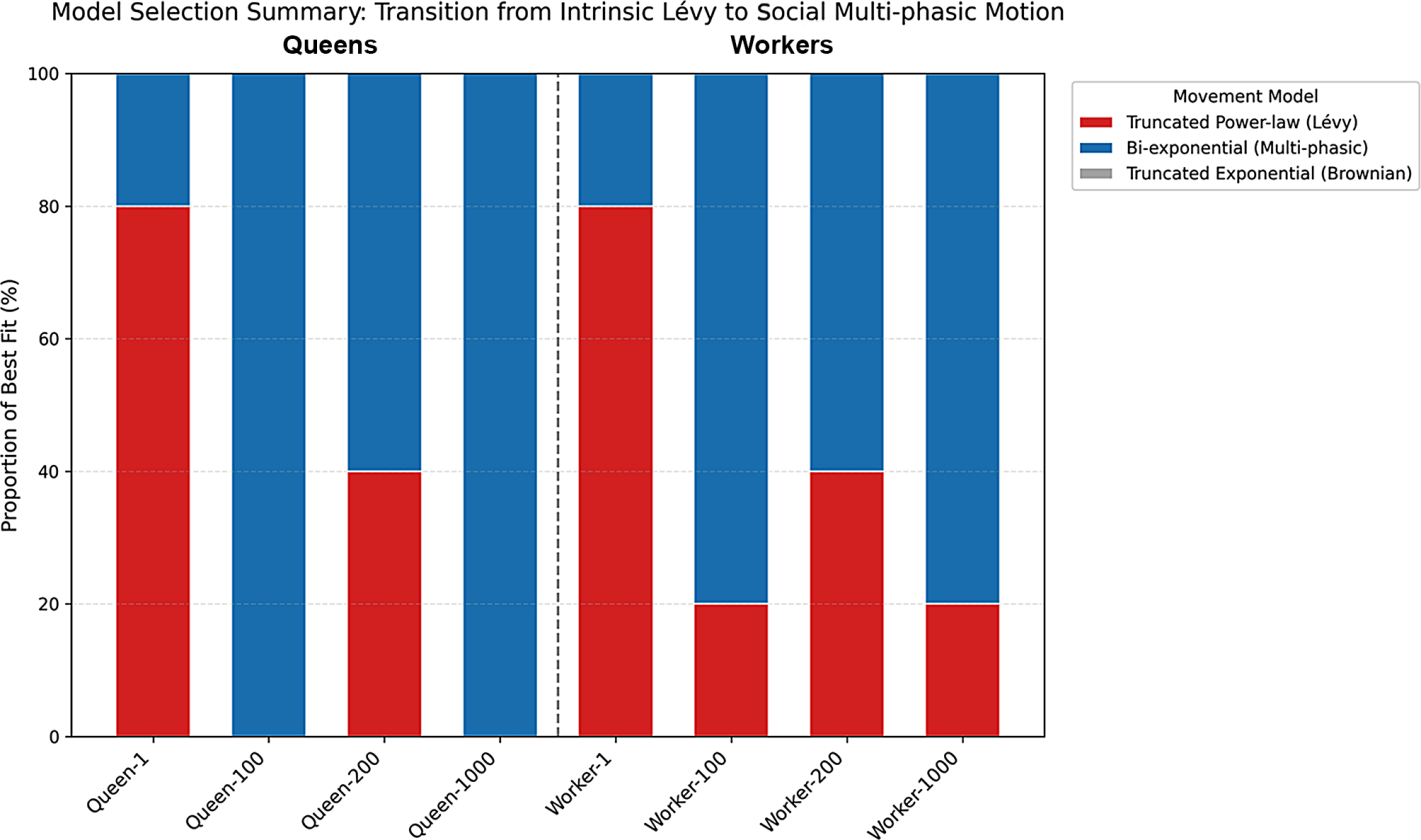
Social crowding drives a transition from intrinsic Lévy walks to multi-phasic movement patterns. Stackced bar chart summarizing the model selection results based on Akaike Information Criterion (AIC) weights for queens and workers across varying group sizes (*N* = 1, 100, 200, 1,000). The height of each bar segment represents the percentage of individuals best fitted by a specific movement model. Red bars (Truncated Power-law, TPL): Represent Lévy-like dynamics. Note that TPL dominates in isolated individuals (*N* = 1), confirming that Lévy walks constitute the intrinsic search template of termites. Blue bars (Bi-exponential, Bi-Exp): Represent multi-phasic (composite Brownian) movements. The dominance of this model in high-density groups (*N* ≥ 100) illustrates how frequent social interactions fragment continuous trajectories into composite phases (e.g., intensive local interaction vs. extensive relocation). Grey bars (Truncated Exponential, TE): Represent simple Brownian motion, which receives minimal support across all social contexts, ruling out pure random walks as the primary driver of movement even under crowding

To identify the statistical nature of termite movement and its modulation by social context, we further analyzed step-length distributions using three competitive models: truncated power-law (TPL), truncated exponential (TE), and bi-exponential (Bi-Exp). Under isolated conditions (*N* = 1), both queens and workers exhibited heavy-tailed step-length distributions best fitted by the TPL model (Fig. 3A, C). AIC weights confirmed that TPL was the winning model for 80% of isolated queens and 80% of isolated workers (Fig. 4). The scaling exponents typically ranged between 1.1 and 1.4, indicating a conserved intrinsic Lévy-like search template in the absence of social interference. As social density increased, a significant shift in model preference occurred (Kruskal-Wallis test on AIC weights: *χ*^*2*^ = 12.8, *P* < 0.01). Visual inspection of complementary cumulative distribution function plots revealed a deviation from TPL in high-density groups (Fig. 3B, D). Quantitative analysis showed that the proportion of individuals best fitted by the Bi-Exp model increased with group size (Fig. 4). Specifically, in groups of 100 or more, the Bi-Exp model become dominant, fitting over 60% of individuals, significantly higher than in the isolated condition (Fisher’s exact test: Odds Ratio = 0.06, *P* < 0.01). This statistical transition confirms that social crowding fragments continuous Lévy trajectories into multi-phasic patterns.

Furthermore, we observed significant differences in movement behaviour between castes. Under solitary conditions (*N* = 1), workers exhibited a significantly lower scaling exponent *µ* compared to queens (*µ* = 1.08 ± 0.03 vs. 1.36 ± 0.04; *t*₈ = 4.903, *P* < 0.01). Crucially, this caste divergence persisted even under high-density conditions. While trajectories for both castes became increasingly restricted as density increased to *N* = 1,000, the nature of this transition differed. For queens, the movement patterns were unanimously best described by the Bi-Exp (100% of samples), while workers retained a degree of heterogeneity, with some individuals still adhering to Truncated Power-Law dynamics even at high densities (Table S1). Quantitatively, the scaling exponent for queens shifted to *µ* = 1.93 ± 0.07, marking a transition to a slower-than-ballistic but faster-than-diffusive regime, whereas workers maintained a significantly lower exponent (*µ* = 1.56 ± 0.08; *t*₈ = 3.704, *P* < 0.01).

### Collision-induced turning dynamics underlie density-dependent movement statistics

**Fig. 5:**
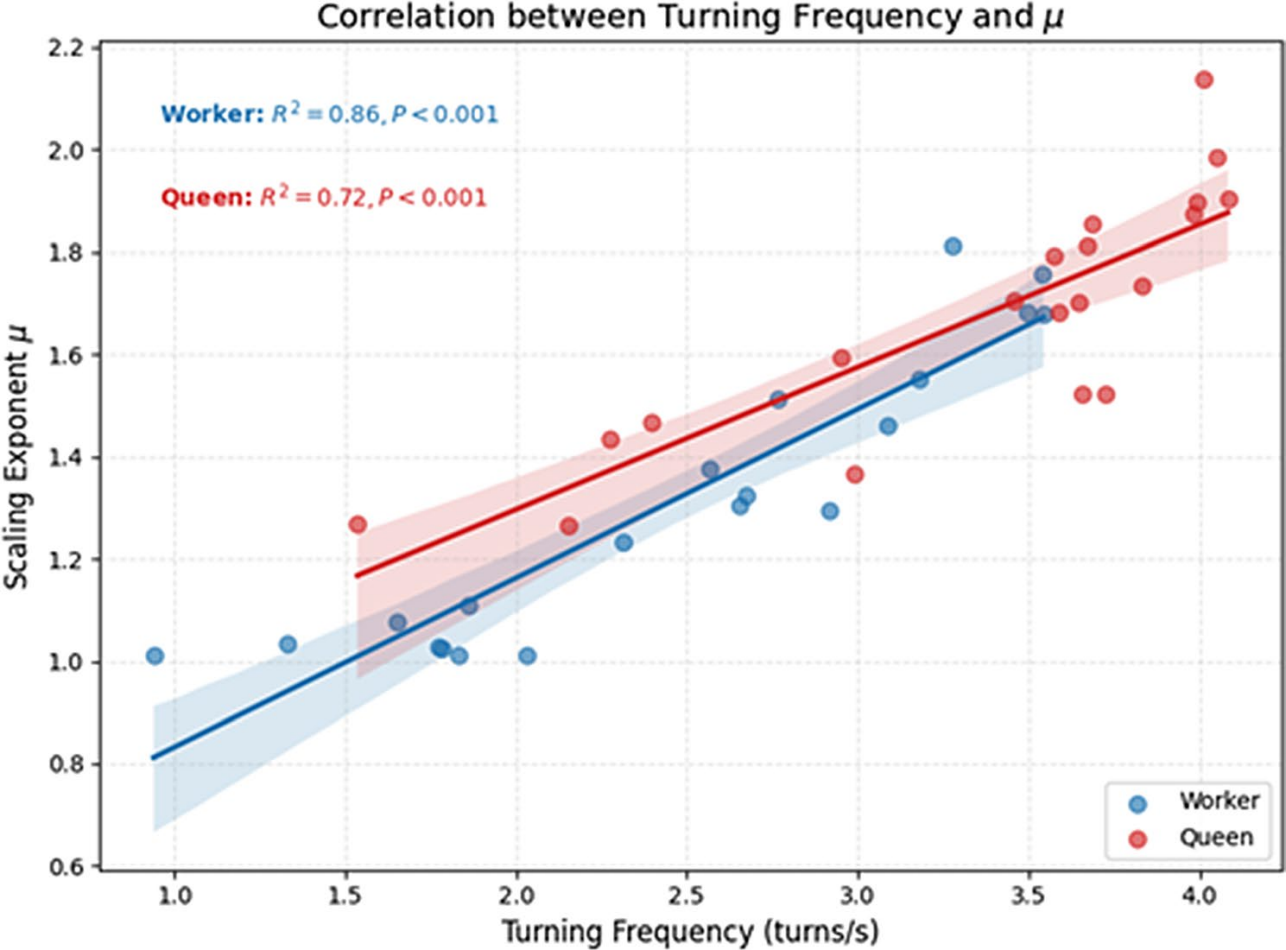
Mechanistic link between individual reorientation behaviour and movement scaling. Linear regression analysis of the relationship between turning frequency (number of turns per second) and the scaling exponent (*µ*) of derived from truncated power-law fits. Data points represent individual queens (red circles) and workers (blue circles) sampled across all density conditions (*N* = 1 to 1,000). Solid lines represent the linear regression fits, and shaded areas indicate the 95% confidence intervals. The strong positive correlation (*R²* = 0.72, *p* < 0.001 for queens; *R²* = 0.86, *p* < 0.001 for workers) demonstrates that the increase in *µ* (i.e., the loss of heavy-tailed scaling) is mechanistically driven by the elevated frequency of reorientations induced by social crowding

To examine whether this density-dependent transition in movement statistics arises from active behavioral modulation or passive physical constraints, we quantified individual turning frequencies and tested their relationship with the scaling exponent *µ* derived from step-length distributions (Fig. 5). Analysis of experimental turning dynamics revealed a strong positive correlation between turning frequency and the *µ*. Linear regression analysis revealed that turning frequency was a significant predictor of *µ* for workers (*R*^*2*^ = 0.86, *P* < 0.001) as well as for queens (*R*^*2*^ = 0.72, *P* < 0.001). This suggests that the steeper power-law scaling statistics at high density may be a direct result of frequent directional interruptions.

**Fig. 6:**
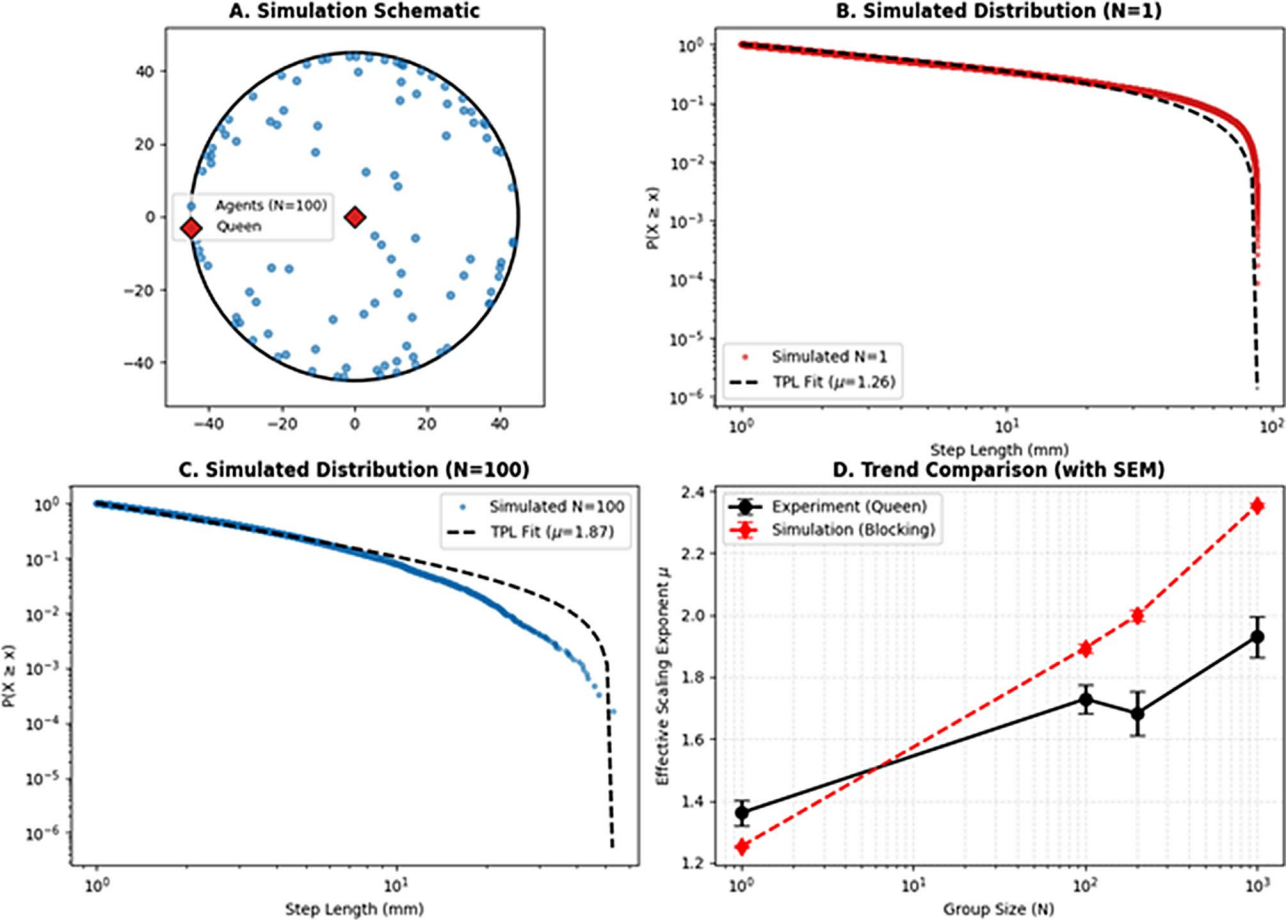
Agent-based simulation validates physical crowding as the driver of movement phase transitions. (**A**) Schematic of the agent-based model setup. Agents are confined within a 90-mm-diameter circular arena. A focal queen (red diamond) is surrounded by workers (blue dots) at varying densities. All agents follow an intrinsic Lévy walk template but are subject to local physical constraints, including boundary truncation and collision-induced blocking. (**B**-**C**) Complementary cumulative distribution functions of simulated step lengths for *N* = 1 (**B**) and *N* = 100 (**C**) on log-log scales. Colored dots represent simulated data, and black dashed lines indicate the best-fit curves derived from the truncated power-law model. (**D**) Quantitative comparison of effective scaling exponents (*µ*) between experimental observations (Queen, black solid line) and simulation results (red dashed line) across group sizes (*N* = 1, 100, 200, 1,000). Error bars represent the standard error of the mean (SEM) for both empirical data and five independent simulation trials. The successful reproduction of the density-dependent increase in *µ* demonstrates that physical blocking and spatial crowding are mechanisms to explain the observed loss of Lévy scaling in social termties

To verify whether simple physical interactions could explain the observed transition, we developed an Agent-Based Model (ABM) parameterized with an intrinsic Lévy exponent (*µ*_*int*_ *=* 1.35) and density-dependent collision rules (Fig. 6). The simulation quantitatively reproduced the experimental results: At *N* = 1, agents exhibited emergent scaling exponents matching the experimental baseline (*µ* ≈ 1.36), confirming that boundary truncation explains the deviation from the theoretical *µ*_*int*_. At *N* = 100, frequent soft collisions fragmented long steps, causing the simulated *µ* to rise to ~ 1.87, closely mirroring the empirical queen data (experimental *µ* ≈ 1.93). These results demonstrate that physical blocking and spatial crowding are mechanisms to generate the observed transition from nearly ballistic to highly restricted superdiffusive motion, without requiring complex cognitive changes.

### Division of labor in exploration and social integration

**Fig. 7:**
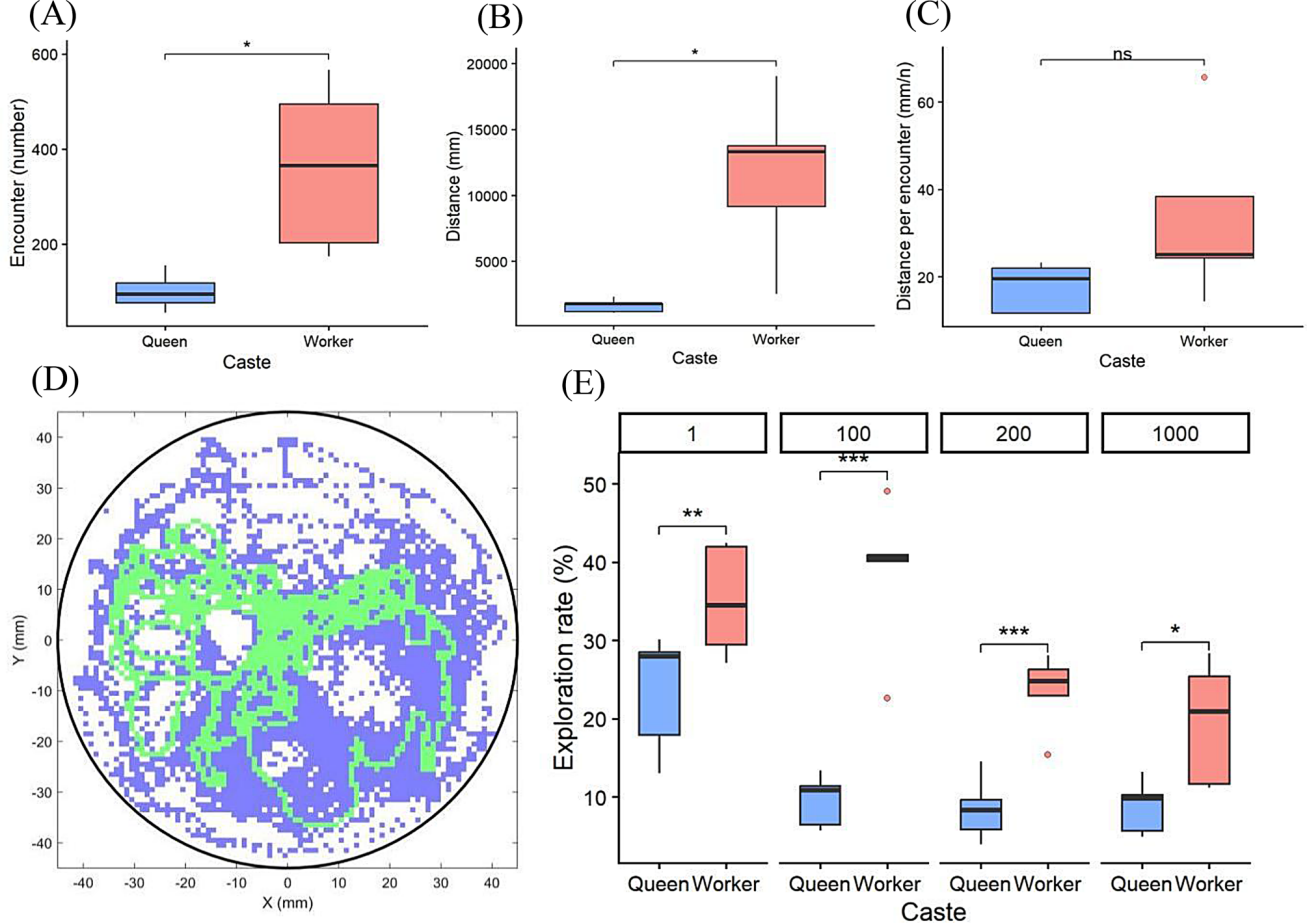
Caste-specific movement behaviours reflect distinct functional roles in termites. (**A**–**C**) Encounter efficiency and movement metrics for queens and workers in groups of 100 individuals. Workers exhibited a significantly higher number of encounters (**A**) and greater total distance traveled (**B**) compared to queens, while the mean distance per encounter (**C**) remained similar across castes. These patterns suggest that workers act as primary social connectors through active locomotion. (**D**) Representative spatial exploration maps illustrating the divergent trajectories of queens (green) and workers (blue). Workers navigated a substantially broader area, whereas queen movement was more localized within the group center. (**E**) Comparison of cumulative exploration rates (%) across group sizes (*N* = 1, 100, 200, 1,000). Workers consistently maintained higher exploration coverage than queens. Boxplots display medians (horizontal lines), interquartile ranges (boxes), and outliers (points). Asterisks denote post hoc Tukey test results (*p < 0.05; **p < 0.01; ***p < 0.001; ns, not significant)

We compared the functional performance of the two castes in 100-individual groups (Fig. 7): Workers achieved higher encounter frequencies (*t*_4_ = 4.24, *P* = 0.013, paired t-test; Fig. 7A) and traveled significantly greater total distances (*t*_4_ = 3.71, *P* = 0.021; Fig. 7B) compared to queens. In addition, we quantified caste-specific differences in exploration rate and spatial coverage across all group-size conditions to assess social density shapes space-use movements in queens and workers. Across all densities, workers consistently explored a larger proportion of the arena than queens. Post hoc comparisons based on estimated marginal means confirmed significant caste differences at each group size (1-individual: *t*_28_ = 2.87, Tukey-adjusted *P* < 0.01; 100-individual: *t*_28_ = 7.21, *P* < 0.001; 200-individual: *t*_28_ = 3.73, *P* < 0.001; 1000-individual: *t*_28_ = 2.66, *P* < 0.05; Fig. 7D, E). Complementary spatial analyses revealed a pronounced caste-dependent pattern of space use. Quantification of inner-region occupancy, analyzed using a generalized linear mixed model with a binomial error distribution and logit link, showed that queens spent significantly greater proportion of time within the inner region than workers across all group-size conditions (1 individual: *z* = 2.79, *P* < 0.01; 100 individuals: *z* = 3.80, *P* < 0.001; 200 individuals: *z* = 5.31, *P* < 0.001; 1,000 individuals: *z* = 4.49, *P* < 0.001; Fig. S2).

To evaluate the functional implications of density-dependent changes in movement scaling, we conducted simulations spanning a range of *µ* from nearly ballistic to slower-than-ballistic superdiffusive regimes. Representative trajectories illustrate that low *µ* values generate long-range ballistic displacements, whereas high *µ* values produce localized, space-filling paths within confined arenas (Figure S3A). Quantification of global search revealed a monotonic decline with increasing *µ.* The number of unique spatial grid cells visited was highest at low *µ* values and progressively decreased as *µ* increased (Figure S3B). In contrast, local interaction potential exhibited the opposite trend. The frequency of path revisits, used as a proxy for localized residence and interaction intensity, increased steadily with *µ* (Figure S3C). These simulations suggest that a functional trade-off mediated by movement scaling: queens adopt a “stay-in-place” behaviour (high *µ*) to promote localized residence and intensified local interactions, whereas workers retain high mobility (low *µ*) to serve as the colony’s mobile connectors and environmental foragers (Figure S3D).

## Discussion

### Density-dependent modulation of intrinsic movement templates

Previous studies on termite movement ecology have largely focused on workers, proposing that Lévy-like patterns are emergent properties driven by social context. Specifically, Paiva et al. (2021) demonstrated that scale-free patterns arise from social interactions via preferential attachment mechanisms [28], and Castiblanco et al. (2022) highlighted that the Lévy exponent scales continuously with group density and worker-soldier composition [44]. While Paiva et al. provided the seminal observation that isolated workers display intrinsic Lévy-like motility, our study expands this framework across the reproductive divide. We demonstrate that solitary queens also possess an intrinsic scale-free template, but operate with a significantly different super-diffusive baseline (*µ* ≈ 1.36) compared to workers (*µ* ≈ 1.08). This quantitative divergence reveals that Lévy movement is not a uniform species-level trait, but a highly heterogeneous, caste-specific behavioral template. This suggests that the Lévy movement is an intrinsic behavioral template, likely encoded for optimal solitary search efficiency in the absence of cues [45]. But this optimal behaviour is not fixed; it is plastically modulated by social density. Under the extreme crowding characteristic of termite natural subterranean tunnel networks, where the confined geometry and high nestmate density inevitably push the system toward a jammed state (as mimicked by our *N* = 1,000 condition), movement patterns universally collapse into multi-phasic, localized dynamics (best fitted by Bi-Exponential models). This transition, statistically confirmed by the significant reduction in path straightness (*P* < 0.01), suggests that the random walk observed in crowded colonies is not a behavioral choice but an emergent property derived from the truncation of intrinsic ballistic runs, consistent with the concept of anomalous diffusion in crowded environments [46].

### Physical blocking as the primary driver of trajectory randomization

Our results demonstrate that social crowding and arena boundaries impose a tempering effect on the intrinsic Lévy motility of termites. As discussed in the foundational works of Mantegna and Stanley [42], pure Lévy stable processes are physically unattainable due to their infinite variance; instead, biological systems often exhibit truncated Lévy walks. By introducing simple steric repulsion (physical blocking) without any assumption of behavioral state changes, our Agent-Based Model successfully reproduced the density-dependent shift in scaling exponents observed empirically (Fig. 6). Paiva et al. previously demonstrated that inert blockages do not disrupt Lévy scaling, elegantly highlighting the primary role of social mechanisms (e.g., preferential attachment) at moderate densities. However, natural subterranean nests are characterized by extreme crowding and confined geometries (as mentioned above). Under such extreme density regimes, our Agent-Based Model demonstrates a critical mechanistic crossover: pervasive physical constraints alone become mechanistically sufficient to drive the transition from scale-free exploration to multi-phasic Brownian dynamics, suggesting that physical mechanics dominate when active social interactions are overwhelmed by spatial limitations. This indicates that physical crowding acts as a low-pass filter for movement, selectively eliminating long-range displacements while preserving short-range jitters. Unlike ant colonies where encounters often stimulate activity via excitability dynamics [47, 48], in the confined tunnels of termites, high density primarily functions as a mechanical constraint that creates a jammed state of localized motion [22]. There are two primary forms of tempering discussed in literature: exponential cutoffs (where *P*(*l*) ~ *l*^−*µ*^*e*^*−λl*^) and steeper power-law truncation (hard cutoffs) [43, 49]. In our system, the transition toward a steepened superdiffusive regime at high densities is best described by the hard truncation imposed by physical blocking. While an exponential cutoff implies a gradual loss of persistence, the frequent and abrupt collisions in termite crowds act as a stochastic hard-reset of the step length, effectively chopping the long-range displacements of the intrinsic template into shorter, multi-phasic segments.

### Caste-specific resilience and functional partitioning

Beyond the general physical constraints, we reveal a crucial functional trade-off driven by caste identity. Workers exhibited a significantly lower intrinsic scaling exponent than queens and, importantly, retained signatures of super-diffusive mobility even under high-density conditions (Table S1). This resilience to crowding equips workers to act as efficient explorers and distributors of resources throughout the colony, optimizing the exploration-exploitation balance [50]. Conversely, queens succumb rapidly to physical entrapment(*µ*→ 1.93). From a statistical physics perspective, for one-sided, positive step-length distributions, *µ* = 2 boundary implies that theoretical threshold where the mean step length becomes finite. Approaching this boundary indicates a transition from an extensive scale-free Lévy regime to a slower-than-ballistic but faster-than-diffusive regime, rather than the onset of Brownian dynamics [51]. This transition for queens highlights their sensitivity to physical entrapment, where social crowding effectively arrests the intrinsic Lévy movement template. By being partially restricted to the colony center, queens minimize energy expenditure and maximize their availability for trophallaxis and grooming, effectively transforming from mobile individuals into static interaction hubs. Thus, the colony leverages the interplay between differential intrinsic motility and uniform physical constraints to spontaneously organize its division of labor [52].

### Broader implications and conclusion

The transition from ballistic to diffusive motion observed here parallels phase transitions in active matter physics, where particle density dictates the shift from fluid-like to glassy dynamics [53, 54]. Our findings suggest that biological systems evolving in confined spaces, from cells in tissues to mole-rats in tunnels, may universally exploit physical crowding to modulate individual behaviour. Future work integrating 3D tracking within natural soil matrices will further elucidate how these statistical laws operate in the complex topology of real nests. In conclusion, termite colonies exemplify how simple physical laws interact with biological adaptations to shape the statistical physics of social life.

## Supplementary Information

Below is the link to the electronic supplementary material.

Supplementary Material 1

Supplementary Material 2

## Data Availability

All data generated or analyzed during this study are included in the supplementary material.
