# Supplementary material for: Physical crowding truncates intrinsic Lévy-like motility into caste-specific movement regimes in social termites
Source: Mov Ecol. 2026 Apr 3;14:31. doi: 10.1186/s40462-026-00646-w (PMC13173929; doi:10.1186/s40462-026-00646-w)
Supplement: Supplementary file 2 — Supplementary Material 2 [file 40462_2026_646_MOESM2_ESM.pdf]

Supplementary Materials

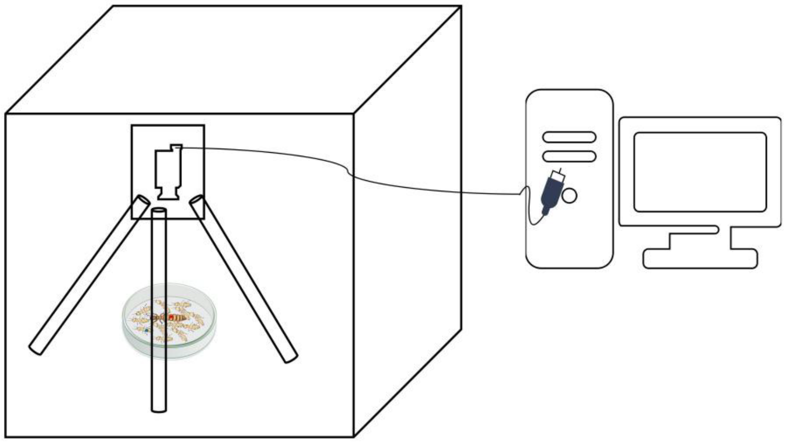

Figure S1. Experimental setup for high-resolution behavioral tracking of termite movement. The recording system consisted of a digital camera mounted above a Petri dish arena inside a controlled observation chamber. The camera was fixed on a tripod stand and connected to a computer for continuous image acquisition and storage.

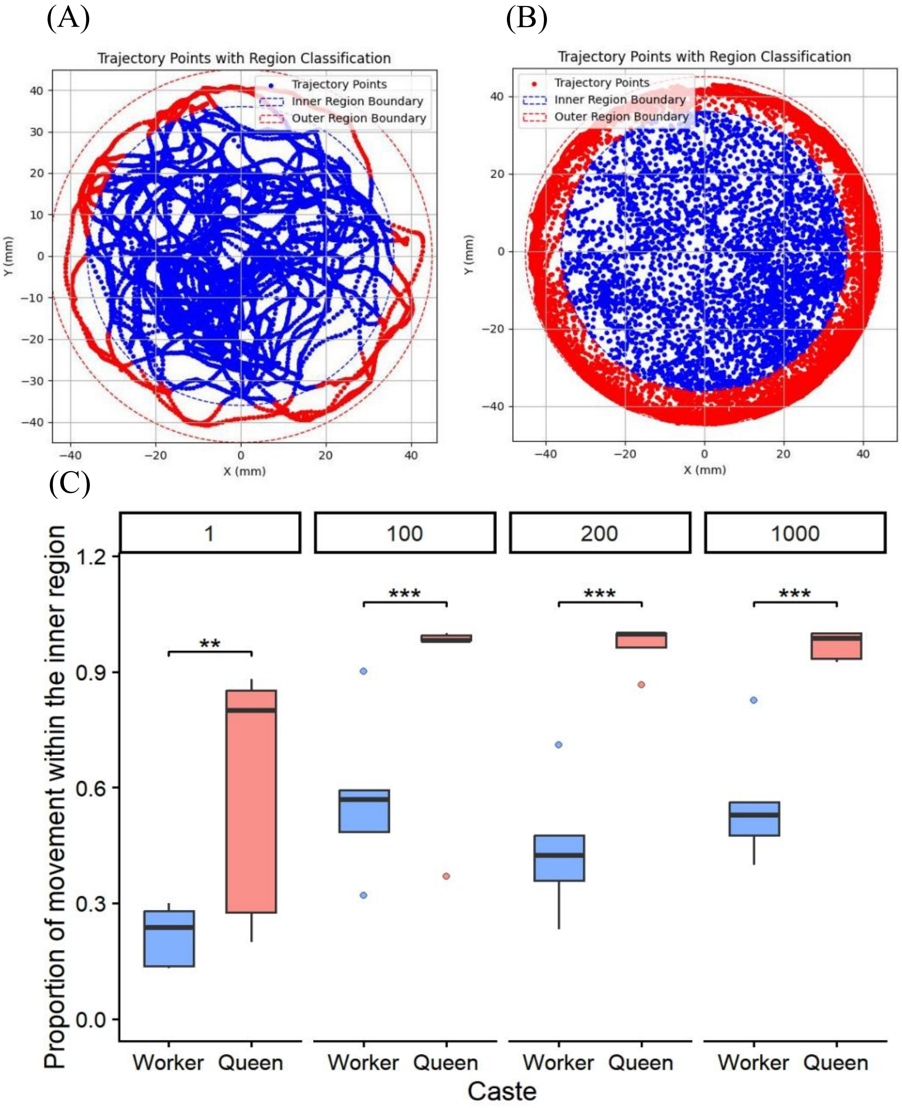

Figure S2. Movement allocation of queens and workers within the arena. (A–B) Representative trajectory maps showing spatial occupancy of queens (A) and workers (B) within a circular arena. Blue and red dots represent trajectory points, while blue and red circles indicate the boundaries of the inner and outer regions used for spatial classification. Queens primarily concentrated their movements in the central area, whereas workers exhibited strong edge-oriented exploration. (C) Proportion of movement occurring within the inner region for queens (red) and workers (blue) across four group sizes (1, 100, 200, and 1,000 individuals). Workers consistently spent less time in the inner region than queens. Boxplots show the median (horizontal line), interquartile range (box), and outliers (points). Asterisks indicate significance levels: $p < 0.01$ (“**”), and $p < 0.001$ (“***”).

.

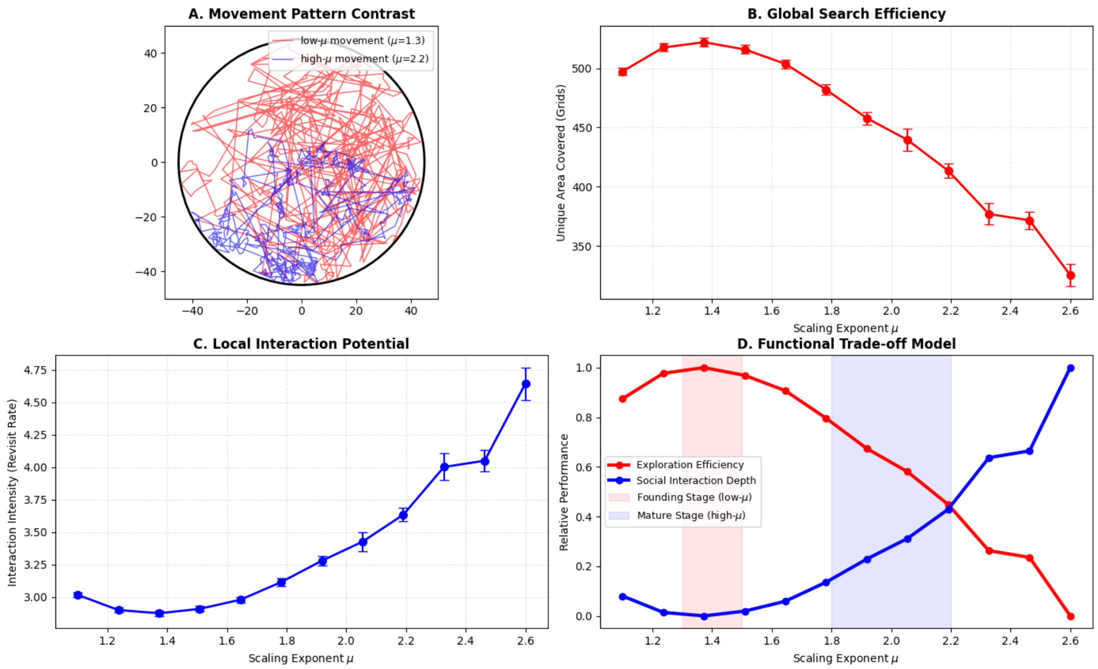

Figure S3. Functional and evolutionary trade-off mediated by scaling exponent modulation. (A) Representative simulated trajectories for a low-μ movement (red) and a high-μ movement (blue) within a confined arena. Low-μ movement exhibits long-range ballistic leaps, whereas the high-μ state is characterized by localized, thorough space-filling. (B) Search efficiency, measured as the number of unique grid cells visited, decreases as μ increases, highlighting the superior exploratory capacity of Lévy walk in sparse environments. (C) Interaction potential (measured as the frequency of local path revisits) increases with μ. High-μ values promote localized residence time, facilitating intensive pheromone exchange and social contact in crowded group centers. (D) A conceptual trade-off model illustrating the transition from a global exploration strategy (low-μ) to a local social interaction strategy (high-μ). This density-dependent phase transition reflects an adaptive strategy where individuals (particularly queens) prioritize social stability and information exchange over search efficiency as the group matures and density increases.

Sample ID	N_Steps	mu (TPL)	AIC (TPL)	Weight (TPL)	lambda (TE)	AIC (TE)	Weight (TE)	w (Bi-Exp)	lambda1 (Bi-Exp)	lambda2 (Bi-Exp)	AIC (Bi-Exp)	Weight (Bi-Exp)	Best Fitting Model
Queen-1-1	340	1.435	1662	0.999	0.161	1921.9	3.561e-57	0.71	0.67	0.057	1675.1	0.001	Truncated Power-law
Queen-1-2	448	1.266	2481.2	0.996	0.134	2695.8	2.438e-47	0.38	1.418	0.087	2492.4	0.004	Truncated Power-law
Queen-1-3	237	1.368	1124.1	0.952	0.194	1253.2	9.212e-29	0.51	1.272	0.104	1130.1	0.048	Truncated Power-law
Queen-1-4	587	1.269	3165.4	0.91	0.152	3383.1	4.757e-48	0.43	0.993	0.093	3170	0.09	Truncated Power-law
Queen-1-5	555	1.468	2611.4	3.173e-04	0.158	3157.9	6.985e-123	0.67	1.019	0.058	2595.3	1	Bi-Exponential
Queen-100-1	650	1.704	2081	0.034	0.448	2345.6	1.175e-59	0.46	2.842	0.26	2074.3	0.966	Bi-Exponential
Queen-100-2	396	1.7	1195.3	0.66	0.544	1276.3	1.643e-18	0.5	1.874	0.318	1196.6	0.34	Bi-Exponential
Queen-100-3	645	1.593	2433.7	2.067e-13	0.396	2486.9	5.888e-25	0.54	1.13	0.223	2375.3	1	Bi-Exponential
Queen-100-4	426	1.856	1169.7	4.733e-05	0.645	1227.4	1.390e-17	0.7	1.377	0.292	1149.8	1	Bi-Exponential
Queen-100-5	500	1.791	1534.6	0.873	0.411	1890.6	4.206e-78	0.6	2.405	0.183	1538.4	0.127	Bi-Exponential
Queen-200-1	523	1.523	2070.8	1	0.292	2334.6	5.103e-58	0.61	1.168	0.135	2098.3	1.039e-06	Truncated Power-law
Queen-200-2	438	1.875	983.8	0.069	0.849	1021	5.659e-10	0.42	2.62	0.572	978.6	0.931	Bi-Exponential
Queen-200-3	661	1.521	2583.3	0.998	0.314	2853.3	2.234e-59	0.57	1.292	0.157	2595.7	0.002	Truncated Power-law
Queen-200-4	655	1.813	1878.9	1.203e-07	0.611	1957.6	1.002e-24	0.48	2.01	0.37	1847	1	Bi-Exponential
Queen-200-5	526	1.682	1520.5	0.001	0.61	1573	4.258e-15	0.57	1.48	0.345	1506.8	0.999	Bi-Exponential
Queen-1000-1	944	1.905	2475.8	2.266e-11	0.686	2602.2	8.244e-39	0.61	1.748	0.355	2426.8	1	Bi-Exponential
Queen-1000-2	938	1.983	2215.6	2.486e-07	0.775	2355.8	9.074e-38	0.61	1.911	0.398	2185.2	1	Bi-Exponential
Queen-1000-3	689	2.138	1204.8	8.564e-07	1.087	1264.5	9.401e-20	0.78	1.828	0.444	1176.9	1	Bi-Exponential
Queen-1000-4	923	1.733	2752.3	1.451e-16	0.604	2779.7	1.609e-22	0.65	1.183	0.315	2679.4	1	Bi-Exponential
Queen-1000-5	543	1.896	1146.3	0.617	0.892	1209.9	9.807e-15	0.51	2.374	0.546	1147.3	0.383	Bi-Exponential
Worker-1-1	861	1.024	5963.2	1	0.048	6807.1	5.521e-184	0.5	0.509	0.027	6152.4	8.200e-42	Truncated Power-law
Worker-1-2	646	1.01	5173.9	1	0.029	5483.1	7.051e-68	0.25	1.003	0.028	5260.2	1.832e-19	Truncated Power-law
Worker-1-3	756	1.233	4454.2	0.219	0.107	4884.9	6.638e-95	0.37	1.578	0.07	4451.7	0.781	Bi-Exponential
Worker-1-4	754	1.035	5189.7	1	0.068	5538.4	1.889e-76	0.36	0.751	0.046	5230.2	1.651e-09	Truncated Power-law

Worker-1-5	750	1.076	5015.6	1	0.065	5569.7	4.935e-121	0.44	0.735	0.038	5084.8	9.396e-16	Truncated Power-law
Worker-100-1	1280	1.028	8703.5	0.11	0.078	9041	5.635e-75	0.24	1.23	0.061	8699.3	0.89	Bi-Exponential
Worker-100-2	1603	1.01	11781	2.551e-105	0.072	11552.6	9.984e-56	0.11	4.144	0.066	11299.3	1	Bi-Exponential
Worker-100-3	981	1.01	6987.1	1	0.061	7373.8	1.035e-84	0.32	0.713	0.044	7043.9	4.516e-13	Truncated Power-law
Worker-100-4	928	1.108	6039.9	0.084	0.086	6399.9	5.650e-80	0.34	0.997	0.059	6035.1	0.916	Bi-Exponential
Worker-100-5	520	1.513	2304.1	0.003	0.204	2694.9	4.615e-88	0.52	1.887	0.105	2292.7	0.997	Bi-Exponential
Worker-200-1	302	1.679	1095.4	1	0.27	1396.2	4.854e-66	0.77	1.071	0.077	1121.4	2.273e-06	Truncated Power-law
Worker-200-2	802	1.375	3900	2.854e-06	0.21	4107	3.153e-51	0.52	0.819	0.116	3874.5	1	Bi-Exponential
Worker-200-3	532	1.296	2841.5	1	0.143	3137.3	5.887e-65	0.47	1.04	0.081	2869	1.082e-06	Truncated Power-law
Worker-200-4	610	1.305	3252.2	1.297e-05	0.163	3436.1	1.474e-45	0.41	1.087	0.102	3229.6	1	Bi-Exponential
Worker-200-5	354	1.812	1050	0.255	0.487	1218.9	5.515e-38	0.76	1.356	0.161	1047.9	0.745	Bi-Exponential
Worker-1000-1	633	1.758	1947	0.367	0.483	2188.3	1.482e-53	0.8	1.084	0.152	1945.9	0.633	Bi-Exponential
Worker-1000-2	731	1.325	3609.2	0.003	0.197	3837	9.375e-53	0.53	0.827	0.107	3597.4	0.997	Bi-exponential
Worker-1000-3	978	1.553	3838.1	4.917e-06	0.321	4181.9	1.128e-80	0.52	1.522	0.173	3813.7	1	Bi-Exponential
Worker-1000-4	408	1.681	1447.4	0.906	0.346	1684.4	3.158e-52	0.62	1.572	0.151	1452	0.094	Truncated Power-law
Worker-1000-5	1022	1.461	4590.7	1.621e-09	0.247	4906	5.381e-78	0.53	1.051	0.132	4550.2	1	Bi-Exponential

35

Table S1. Detailed results of statistical model fitting for termite step-length distribution across castes and group sizes. This table provides a comprehensive summary of parameter estimates and model selection metrics for three candidate movement models: the Truncated Power-law (TPL), Truncated Exponential (TE), and Bi-exponential (Bi-Exp) mixture model. For each experimental sample, the scaling exponent (μ for TPL), rate parameters (λ, λ_1, λ_2), and mixing weights (w for Bi-Exp) were estimated using Maximum Likelihood Estimation (MLE). Model performance was evaluated using the Akaike Information Criterion (AIC) and Akaike weights (W), where the "Best Fitting Model" corresponds to the distribution with the lowest AIC value. The sample size (N) denotes the total number of steps analyzed for each individual queen or worker across different group sizes (1, 100, 200, and 1,000 individuals).

Supplementary Methods

Simulation of functional trade-offs between exploration and interaction

To evaluate the functional consequences of the observed motility transition, we performed numerical simulations of agents exhibiting truncated Lévy walks with varying scaling exponents $\mu \in [1.1, 2.6]$. The simulation environment was a circular arena (R = 45 units) mirroring the experimental setup.

Movement model: Step lengths (l) were generated using inverse transform sampling from a power-law distribution:

$$l = l_{min} \cdot (1-u)^{\frac{1}{1-\mu}}$$

Where $u \sim U(0,1)$ is a uniform random variable, and l_{min} = 1.0 units. To mimic physical constraints, step lengths were capped at $0.8R$. Agents moved in random directions ($(\theta \sim [0, 2\pi))$). Boundary conditions were handled by enforcing a stop-and-wait rule if a step projected the agent outside the arena.

Functional metrics: We simulated trajectories of 1,500 steps for each μ value (10 independent replicates per μ) and calculated two complementary functional metrics based on a grid discretization method (grid resolution = 3.0 units):

1. Global search efficiency: Defined as the total count of unique grid cells visited by the agent. High values indicate extensive spatial coverage (exploration).

2. Local interaction potential: Defined as the revisit rate (total steps / unique grid cells). Higher values imply that the agent repeatedly traverses the same local area, thereby increasing the probability of encountering nestmates or brood (intensification).

Statistical analysis: Mean values and standard error of the mean (SEM) were calculated for both metrics across the range of μ. To visualize the functional trade-off (Figure S3D), both metrics were min-max normalized to a range of [0, 1]. The intersection of these trends illustrates the shift from an exploration-dominated strategy (low μ, typical of solitary founders) to an interaction-dominated strategy (high μ, typical of mature colonies).
